# Comparable outcomes and early revision rates between restricted and unrestricted functional knee positioning in robotic‐assisted total knee arthroplasty for varus deformities ≥10°

**DOI:** 10.1002/ksa.70055

**Published:** 2025-08-31

**Authors:** Christos Koutserimpas, Konstantinos Dretakis, Enejd Veizi, Nevzat Arıcan, Elvire Servien, Cécile Batailler, Sébastien Lustig

**Affiliations:** ^1^ Department of Orthopaedics Surgery and Sports Medicine FIFA Medical Center of Excellence, Croix‐Rousse Hospital, Lyon University Hospital, Hospices Civils de Lyon Lyon France; ^2^ School of Rehabilitation Health Sciences University of Patras Patras Greece; ^3^ 2nd Department of Orthopaedics “Hygeia” General Hospital of Athens Athens Greece; ^4^ Department of Orthopedics and Traumatology Ankara Bilkent City Hospital Ankara Turkey; ^5^ LIBM‐EA 7424, Interuniversity Laboratory of Biology of Mobility Claude Bernard Lyon 1 University Lyon France; ^6^ Univ Lyon, Claude Bernard Lyon 1 University, IFSTTAR, LBMC UMR_T9406 Lyon France

**Keywords:** personalised alignment, robotic knee, robotic‐assisted knee, TKA, total knee

## Abstract

**Purpose:**

Functional knee positioning (FKPos) in total knee arthroplasty (TKA) optimises outcomes by balancing individual anatomical and soft tissue characteristics. Managing marked varus deformity presents challenges in achieving balance when tibial alignment is restricted to 3° of varus, necessitating either medial soft tissue release or unrestricted tibial positioning. This study aims to compare restricted FKPos with medial soft tissue release to unrestricted FKPos without soft tissue release in patients with varus deformity ≥10**°**.

**Methods:**

This retrospective, two‐center study analysed robotic‐assisted TKAs. Patients were categorised into two groups: restricted FKPos with medial soft tissue release (Group A) and unrestricted FKPos without soft tissue release (Group B). Inclusion criteria required a preoperative coronal alignment of ≥10° varus. Outcomes included Knee Society Scores (KSS), Forgotten Joint Scores (FJS), complications, and implant survivorship over a median follow‐up of 38 months (interquartile range [IQR] 30–45).

**Results:**

A total of 205 patients (Group A: 71 and Group B: 134) were included. No significant differences were observed in functional outcomes (KSS and FJS) or complication rates between the groups. Median tibial varus alignment was 1° in Group A and 4.5° in Group B (*p* < 0.0001). Implant survivorship was comparable between groups (1.4% in Group A and 0.75% in Group B; *p* = 0.65; hazard ratio = 1.94; 95% confidence interval: 0.11–35.62).

**Conclusion:**

This study demonstrated that restricted FKPos with medial soft tissue release and unrestricted FKPos without soft tissue release result in comparable short‐term outcomes, complication rates, and implant survivorship in ≥10° varus deformities. While these findings suggest that unrestricted tibial positioning may be a promising alternative to traditional approaches, further studies with long‐term follow‐up are needed to confirm its safety and durability.

**Level of Evidence:**

Level III.

**Abbreviations**
BMI: body mass index
CI: confidence interval
CT: computed tomography
FA: functional alignment
FJS: Forgotten Joint Score
FKPos: functional knee positioning
HKA: hip‐knee‐ankle angle
HR: hazard ratio
IQR: interquartile range
KSS: Knee Society Score
SD: standard deviation
TKA: total knee arthroplasty

## INTRODUCTION

Functional alignment (FA), or functional knee positioning (FKPos), is a personalised strategy in total knee arthroplasty (TKA) that balances bony structures and soft tissue tension in three dimensions, based on mediolateral gap assessment in knee extension and at 90° flexion [1, 2, 3, 4, 9, 15, 26, 30]. Robotic systems provide real‐time intraoperative data to optimise implant positioning, adapting to patient anatomy and ligament behaviour while maintaining accuracy and safety [8, 17, 24]. Despite promising results, FA remains a relatively new strategy with limited but growing outcome data [5, 6, 13, 14, 16, 20, 21].

In marked varus knees, FKPos is challenging when tibial alignment is limited to 3° varus, as this may hinder gap balancing and require medial soft tissue release to reduce tension [11, 27]. Exceeding 3° can better restore native alignment and reduce releases in select cases, though its effects remain under investigation [18]. This contrast underscores a key issue in FKPos for varus knees.

This two‐center comparative study (minimum 2‐year follow‐up) evaluates restricted FKPos with medial soft tissue release versus unrestricted FKPos without release in patients with varus deformity ≥ 10° undergoing robotic‐assisted TKA. It examines whether exceeding the conventional 3° tibial varus limit achieves outcomes comparable to restricted alignment with release. Primary outcomes are implant survivorship, complication rates and functional scores.

## METHODS

This study is a retrospective comparative analysis of robotic‐assisted TKAs performed according to FKPos principles at two high‐volume institutions specialising in image‐based robotic surgery. The study evaluates data from two prospectively maintained databases: the 2nd Department of Orthopaedics at Hygeia Hospital, Athens, Greece (Group A), and the Orthopaedics Surgery and Sports Medicine Department at University Hospital Croix Rousse, Lyon, France (Group B). Both institutions utilise the Mako Robotic‐Arm Assisted Surgery System (Stryker, Mako Surgical Corp., Fort Lauderdale, FL, USA). This robotic platform combines preoperative computed tomography (CT) imaging with intraoperative haptic guidance, allowing surgeons to create a highly detailed, patient‐specific 3D plan for precise implant positioning.

In the Group A, a restricted tibial varus alignment of up to 3° was employed. When required, medial soft tissue releases were performed to achieve balance within these boundaries. Conversely, in the Group B, tibial varus positioning was permitted up to 6°, a threshold referred to in recent literature as 'unrestricted tibial positioning', as opposed to the more traditional restriction of ≤ 3° [11, 18]. No soft tissue releases were performed in group B. This dual‐center design facilitates a direct comparison between two distinct approaches to FKPos: one prioritising soft tissue balancing within restricted alignment boundaries and the other emphasising unrestricted anatomical alignment without tissue manipulation.

The databases were utilised from their inception (06/2020 for Group A and 03/2021 for Group B) up to 01/2023. The minimum follow‐up of this study was 2 years. All patients received either a CR or a PS fixed‐bearing implant (Triathlon, Stryker, MI, USA), with PS implants selected for cases involving posterior cruciate ligament insufficiency or significant flexion contractures. Both insert types have exhibited similar outcomes in FKPos [19].

Only patients with preoperative coronal alignment ≥10° varus (robotic data) undergoing robotic‐assisted TKA with FKPos were included. This threshold, identified in Group A where all releases occurred at ≥10° varus, was applied to Group B for consistency.

Valgus and revision cases, patients with inadequate follow‐up or those undergoing TKA under the mechanical alignment principles due to prior trauma, soft tissue and ligamentous instabilities were excluded.

Patient demographics, including age, sex and body mass index (BMI), were recorded. Patients were categorised into two main groups based on the alignment strategy employed: restricted FKPos with medial soft tissue release (Group A) and unrestricted FKPos without soft tissue release (Group B). All procedures followed the principles of FKPos, a three‐dimensional, patient‐specific alignment philosophy utilising robotic‐assisted technology to optimise both tibiofemoral and patellofemoral kinematics [15]. In group A, the tibial implant was limited to a maximum of 3° varus. After osteophyte removal and stress testing, alignment and gap balance were dynamically assessed. When gaps remained unbalanced despite permissible component adjustments, a stepwise release of the superficial medial collateral ligament (MCL) and posteromedial capsule was performed using a pie‐crusting technique or subperiosteal release as needed [11]. In contrast, Group B permitted tibial alignment up to 6° varus, allowing restoration of the patient's constitutional anatomy without soft tissue manipulation [15, 18]. The femoral and tibial component positions were iteratively adjusted intraoperatively in all planes to achieve balanced gaps, with a target of 1–2 mm lateral laxity in flexion and extension. Implant design (CR vs. PS) was selected based on posterior cruciate ligament integrity and presence of flexion contracture. All surgeries were performed by experienced surgeons trained in robotic‐assisted FKPos, with one lead surgeon per center to ensure consistency.

In both centres, all surgeries used a standard midvastus approach without a tourniquet. Regional (spinal) anaesthesia was preferred in most cases. Postoperative thromboprophylaxis was given with low‐molecular‐weight heparin. All patients received periarticular multimodal infiltration (ropivacaine, ketorolac and epinephrine) to the posterior capsule, medial/lateral gutters, and peri‐incisional tissues before closure. Rehabilitation protocols were similar, with early mobilisation on the day of or first day after surgery, full weight‐bearing as tolerated and structured physiotherapy for range of motion, quadriceps activation, gait training and progressive strengthening.

Pre‐ and postoperative coronal alignment was measured as the mechanical hip–knee–ankle (HKA) angle in extension using the robotic system, which also provided implant positioning data. Clinical outcomes were assessed preoperatively and at final follow‐up with Knee Society Score (KSS)‐knee and‐function and the Forgotten Joint Score (FJS). Complications, including minor events (e.g., haematomas) and major adverse events requiring surgery, as well as revisions, were recorded and compared between groups.

### Statistical analyses

The Kolmogorov–Smirnov test was utilised to evaluate the normality of data distributions. Depending on the characteristics of the data, group comparisons were conducted using either the independent t‐test for normally distributed variables or the Mann–Whitney *U* test for non‐normal distributions. The Chi‐square test was employed to compare complication rates between the groups. A *p*‐value of less than 0.05 was considered statistically significant. Implant survival was analysed using the Kaplan–Meier method. Multivariate regression analysis was performed, including the following variables: tibial positioning (≤3° [Group A] and >3° of varus [Group B]), age, BMI, gender, preoperative KSS‐ knee and function, tibial and femoral prostheses positioning and combined varus of the femoral and tibial implants. All statistical analyses were performed using MedCalc software (version 22.021). A post hoc power analysis was performed using Gpower software v.3.1.9.8 for the revision rates between the two groups using a two‐tailed Fisher's exact test, assuming *α* = 0.05 and respective group sizes (*N* = 71 and *N* = 134).

### Ethical approval

This study was conducted in compliance with the ethical standards established by the institutional and national research committees, adhering to the principles outlined in the 1964 Declaration of Helsinki and its subsequent amendments or equivalent ethical guidelines. Data collection and analysis followed the MR004 Reference Methodology from the Commission Nationale de l'Informatique et des Libertés (Reference No. 2229975V0). Additionally, the study received ethical approval from the Scientific Committee of Hygeia Hospital in Athens (Reference No. 663, 20/12/2023). Given the retrospective design of the study and the use of anonymized data, the requirement for formal patient consent was waived in accordance with institutional policies.

## RESULTS

### Patient demographics and preoperative evaluation

**Figure 1:**
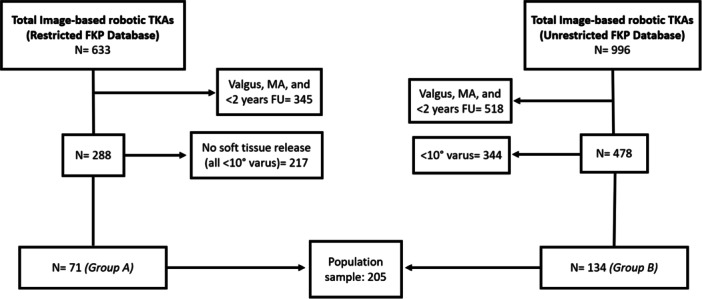
Flowchart of the study selection process. FKP, Functional knee positioning; MA, Mako alignment; TKA, total knee arthroplasty.

A total of 205 patients (54.63% females) with mean age = 71.37 years (standard deviation [SD] = 6.71] and mean BMI = 28.36 kg/m^2^ (SD = 4.81) and a median follow‐up of 38 months (interquartile range [IQR] 30–45) were included for final analysis (Figure 1).

A total of 205 patients were included (Group A: 71; Group B: 134). There were no significant differences in age, sex, or BMI between groups. Preoperatively, Group A showed significantly median lower KSS–Function scores (*p* = 0.01), while median coronal alignment was comparable (*p* = 0.12) (Table 1).

**Table 1:** Comparative analysis of the demographics, the preoperative clinical evaluation and coronal alignment.

	Group A (*N* = 71)	Group B (*N* = 134)	*p*‐value
Demographics			
Age (years)	72.34 (SD = 6.46)	70.85 (SD = 6.81)	0.63
Female gender	46.48%	58.96%	0.09
BMI (kg/m^2^)	27.72 (SD = 4.94)	28.69 (SD = 4.73)	0.17
Preoperative evaluation			
KSS‐knee	62.01 (SD = 12.74)	62.48 (SD = 13.78)	0.48
KSS‐function	61 (IQR 50–70)	70 (IQR 60–80)	**0.01**
Preoperative Mako Alignment (varus)	12° (IQR 10–14)	11° (IQR 10–15)	0.12

*Note*: Group A: Restricted functional knee positioning and medial soft tissue release, group B: Unrestricted functional knee positioning without soft tissue release. Statistically significant values are presented in bold.

Abbreviations: BMI, body mass index; IQR, interquartile range; KSS, Knee Society Score; *N*, number; SD, standard deviation.

### Tibial and femoral implant positioning

Implant positioning differed significantly between groups. Group A showed a median tibial varus of 1° compared to 4.5° in Group B. Femoral valgus was −2.1° in Group A and −1.1° in Group B, while femoral external rotation was greater in Group A (1.8° vs. 0.4°). Combined femoral and tibial varus was also lower in Group A (3.1° vs. 5°). All these differences were statistically significant (*p* < 0.0001) (Table 2).

**Table 2:** Femoral and tibial implant positioning during the robotic‐assisted total knee arthroplasty under the functional alignment principles.

	Group A (*N* = 71)	Group B (*N* = 134)	*p*‐value
Tibial varus	1° (IQR 0.25–2)	4.5° (IQR 3.5–5)	**<0.0001**
Tibial posterior slope	1° (IQR 1–3)	1° (IQR 0–1)	**<0.0001**
Femoral external rotation (reference TEA)	1.8° (IQR 0.9–2.78)	0.4° (IQR −1.1 to 1.43)	**<0.0001**
Femoral valgus	−2.1° (IQR −2.3 to −1.1)	−1.1° (IQR −2 to 0.5)	**<0.0001**
Femoral flexion	4.4° (IQR 2.9–5)	7° (IQR 4.98–8.83)	**<0.0001**
Combined varus (femoral + tibial implant)	3.1° (IQR 2.2–4.2)	5° (IQR 3.8–6.8)	**<0.0001**

*Note*: Group A: Restricted functional knee positioning and medial soft tissue release, group B: Unrestricted functional knee positioning without soft tissue release. Statistically significant values are presented in bold.

Abbreviations: CR, cruciate retaining; PS, posterior stabilised; TEA, trans‐epicondylar axis.

### Postoperative alignment

Postoperative coronal alignment in extension (varus) measured by the robotic system was significantly lower in Group A (median: 5°) compared to Group B (median: 6°; *p* = 0.0001).

### Clinical outcomes

At a median follow‐up of 38 months, no significant differences were observed in clinical outcomes. Median KSS‐Knee was 92 in Group A and 94 in Group B (*p* = 0.53), while KSS‐Function was 90 in both groups (*p* = 0.83). The FJS was also comparable (88 vs. 86; *p* = 0.29) (Table 3).

**Table 3:** Postoperative evaluation of the two groups.

	Group A (*N* = 71)	Group B (*N* = 134)	*p*‐value
Postoperative evaluation			
KSS‐ knee	92 (IQR 90–100)	94 (IQR 90–100)	0.53
KSS‐ function	90 (IQR 90–100)	90 (IQR 90–100)	0.83
FJS	88 (IQR 79.75–94)	86 (IQR 71.25– 92)	0.29
Postoperative Mako Alignment (varus)	5° (IQR 3–6)	6° (IQR 5–7)	**0.0001**

*Note*: Group A: Restricted functional knee positioning and medial soft tissue release, group B: Unrestricted functional knee positioning without soft tissue release. Statistically significant values are presented in bold.

Abbreviations: FJS, Forgotten Joint Score; IQR, interquartile range; KSS, Knee Society Score; *N*, number.

### Complications and revisions

The overall complication rate was similar between groups: 14.1% in Group A and 13.4% in Group B (*p* = 0.9). These included all reported events, with haematomas being the most frequent; none of which required surgical intervention. Only one revision occurred in each group (1.4% vs. 0.75%, *p* = 0.65), and no additional causes for implant failure were recorded (Table 4).

**Table 4:** Complications and revisions are presented in a comparative manner between the two groups.

Complications and revisions	Group A (*N* = 71)	Group B (*N* = 134)	*p*‐value
Total complications	14.08% (*N* = 10)	13.43% (*N* = 18)	0.9
All‐cause revisions	1.4% (*N* = 1)	0.75% (*N* = 1)	0.65
Revisions (mechanical aetiology)	–	–	–
Infections‐DAIR or two‐stage exchange arthroplasty	1.4% (*N* = 1)	0.75% (*N* = 1)	0.65
MUA	2.82% (*N* = 2)	5.97% (*N* = 8)	0.32
Haematoma	9.86% (*N* = 7)	6.72% (*N* = 9)	0.43

*Note*: Group A: Restricted functional knee positioning and medial soft tissue release, group B: Unrestricted functional knee positioning without soft tissue release.

Abbreviations: DAIR, debridement, antibiotics, implant retention; MUA, manipulation under anaesthesia; *N*, number.

**Figure 2:**
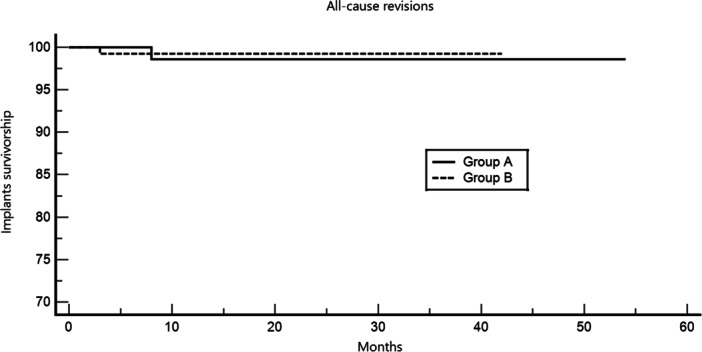
Kaplan–Meier survival analysis comparing all‐cause implant revision between Group A and Group B. No significant difference in implant survivorship was observed between groups (*p* = 0.65; HR = 1.94; 95% CI: 0.11–35.62). CI, confidence interval; HR, hazard ratio.

The post‐hoc power analysis on the comparison of the two groups with regard to revision rates (1.4% vs. 0.75%) showed a power of 99.12%. Kaplan–Meier survival analysis showed no significant difference in implant survivorship between the groups (*p* = 0.65), with hazard ratio (HR) = 1.94; 95% confidence interval (CI): 0.11–35.62 (Figure 2).

Multivariate regression analysis showed that none of the included covariates (tibial positioning [≤3° {Group A} and >3° of varus {Group B}]), age, BMI, gender, preoperative KSS‐knee and function, tibial and femoral prostheses positioning and combined varus of the femoral and tibial implants} were statistically significant predictors of implant failure (Table 5).

**Table 5:** Cox regression with endpoint implant failure showed that none of the included covariates (tibial positioning [≤3° {Group A} and >3° of varus {Group B}], age, BMI, gender, preoperative KSS‐knee and function, tibial and femoral prostheses positioning and combined varus of the femoral and tibial implants) were statistically significant predictors of implant failure statistically significant values are presented in bold.

Covariate	Hazard ratio (HR)	95% CI of HR	*p*‐Value
Restricted functional knee positioning and medial soft tissue release (Group A), Unrestricted functional knee positioning without soft tissue release (Group B.	1	0 to 10.1423E + 303	0.9898
Gender (M/F)	16.3956	0 to 10.1423E + 303	0.9972
Age	0.6405	3.1953E‐52 to 1.284E + 51	0.9941
Preoperative KSS‐function	2.5171	3.2976E‐24 to 1.9213E + 24	0.9738
Preoperative KSS‐knee	1.1071	5.4822E‐27 to 223.5823E + 24	0.9974
Tibial prosthesis varus	1	3.4857E‐248 to 398.0494E + 249	0.9870
Tibial prosthesis posterior slope	0.0002	0 to 10.1423E + 303	0.9901
Femoral prosthesis valgus	0.0040	0 to 10.1423E + 303	0.9921
Femoral prosthesis external rotation	0.0865	5.2839E‐233 to 141.7622E + 228	0.9928
Femoral prosthesis flexion	0.7750	1.8614E‐157 to 3.2269E + 156	0.9989
Combined (femoral and tibial prostheses) varus	5.7299	3.0322E‐284 to 1.0828E + 285	0.9958

Abbreviations: BMI, body mass index; CI, confidence interval; HR, hazard ratio.

## DISCUSSION

This study represents the first direct comparison of two distinct strategies for managing varus deformity ≥ 10° in robotic‐assisted TKA using FKPos principles: restricted tibial positioning (≤3° of varus) with medial soft tissue release and unrestricted tibial positioning (up to 6° of varus) without soft tissue release. The main findings of the study demonstrate that both strategies yield comparable final functional outcomes, as assessed by the KSS and FJS, and show similar rates of complications and revisions over a median follow‐up period of 38 months. These results suggest that expanding the tibial varus alignment beyond 3° without soft tissue release may be a viable alternative to the more traditional approach of restricted tibial alignment with soft tissue balancing in severe varus cases. The comparable survivorship and clinical outcomes observed in this study provide valuable evidence supporting the safety and efficacy of both strategies.

Preoperatively, Group A had a lower median KSS‐function than Group B (61 vs. 70; *p* = 0.01), possibly reflecting cultural and lifestyle differences. Prior studies suggest such variations may relate to differences in healthcare‐seeking behaviour, pain perception and preoperative activity levels [12, 25, 29]. Although cultural factors were not assessed, differences in perceived function and healthcare use may have contributed to the preoperative disparity. However, alignment parameters, KSS–knee scores, and demographics (age, sex and BMI) were similar, indicating comparable cohorts.

Implant positioning differences reflected the effect of tibial alignment limits on three‐dimensional orientation. In Group A (≤3° tibial varus), greater femoral varus and external rotation were used to balance mediolateral gaps in extension and flexion, consistent with FKPos principles of soft tissue–driven adjustment [5, 6, 7, 10, 23]. Within the FKPos framework, limiting tibial alignment requires compensatory femoral adjustments to preserve soft tissue balance. Group B's more varus final alignment reflects unrestricted tibial positioning, avoiding releases and maintaining alignment closer to native anatomy. Recent studies similarly suggest that severe deformities should not be overcorrected with personalised alignment [5, 6, 12, 25]. It should also be taken into consideration that all the included cases were knee with ≥ 10° of varus (from the robotic‐system alignment), while positioning the femoral component in up to 3° of varus does not seem to lead to higher revision rates [22, 28, 31].

The present study demonstrated no statistically significant differences in clinical outcomes, between the two groups at the final follow‐up (median 38 months). These findings suggest that both strategies (restricted FKPos with medial soft tissue release and unrestricted FKPos without soft tissue release) are effective in achieving comparable functional outcomes for patients with ≥10° varus deformity undergoing robotic‐assisted TKA. The comparable outcomes align with prior evidence demonstrating that soft tissue releases, when performed judiciously, do not result in inferior results within restricted alignment boundaries [11]. However, the present study extends this understanding by showing similar results in unrestricted tibial positioning, providing evidence for its utility in managing cases with ≥10° of varus. It should be noted that unrestricted tibial positioning (up to 6° of varus) has been proven safe in the short term with similar functional outcomes and implants' survivorship when compared to tibial positioning of <3° of varus [18].

This study sheds light on the FKPos strategy by comparing two distinct approaches: restricted alignment with soft tissue release and unrestricted tibial positioning without release. Beyond this technical comparison, it also adds to the growing evidence supporting functional alignment in varus knees, with outcomes consistent with previously reported short‐term results, typically up to two years of follow‐up [6, 7, 13, 21, 23].

Complication rates and revision outcomes were similar between groups, with no significant difference in implant survivorship over the 38‐month median follow‐up. Kaplan–Meier analysis supported the short‐term safety of both approaches, and multivariate analysis found no predictors of implant failure among prosthesis positioning, age, BMI, gender or preoperative KSS scores. Most complications were minor, with haematomas being the most frequent and none requiring surgery. One revision occurred in each group (1.4% in Group A; 0.75% in Group B), both due to infection, and no mechanical failures were noted. Longer follow‐up is needed to assess survivorship differences.

This study has some limitations. As a retrospective analysis, it is subject to inherent biases associated with observational study designs. While the follow‐up period of 38 months is adequate to evaluate early outcomes, it is not sufficient to assess long‐term implant survivorship and complications. A significant limitation is the absence of standardised postoperative radiological evaluation. As such, we were unable to radiographically assess component alignment or detect potential early signs of implant migration or loosening. This is particularly relevant in the unrestricted tibial varus group, where positioning the tibial component beyond 3° of varus could be associated with concerns about increased stress at the bone–implant interface and theoretical risk for long‐term aseptic loosening. Although the current findings suggest comparable early outcomes between restricted and unrestricted FKPos strategies, these results should be interpreted with caution, and further studies incorporating long‐term follow‐up and radiographic surveillance are necessary to validate the safety and durability of unrestricted tibial alignment. Despite these limitations, this study has notable strengths. It is a dual‐center analysis with a large cohort of patients undergoing robotic‐assisted TKA, enhancing its generalisability. Furthermore, this is the first study to directly compare restricted FKPos with soft tissue release to unrestricted FKPos without soft tissue release in patients with ≥10° of varus deformity, providing valuable insights into these two distinct strategies within the spectrum of FKPos.

## CONCLUSION

Restricted FKPos with medial soft tissue release and unrestricted FKPos without release resulted in comparable functional outcomes, complication rates, and implant survivorship at a median 38‐month follow‐up in patients with ≥10° of varus deformity undergoing image‐based robotic‐assisted TKA. These short‐term results suggest that unrestricted tibial positioning may be a viable alternative to traditional alignment strategies involving soft tissue balancing. However, longer‐term follow‐up is warranted to confirm the durability of these findings.

## AUTHOR CONTRIBUTIONS

*Conceptualisation*: Christos Koutserimpas, Konstantinos Dretakis, Elvire Servien, and Sébastien Lustig. *Data curation*: Christos Koutserimpas, Enejd Veizi, Nevzat Arıcan and Cécile Batailler. *Formal analysis*: Christos Koutserimpas, Konstantinos Dretakis, Enejd Veizi, Nevzat Arıcan and Cécile Batailler. *Investigation*: Konstantinos Dretakis, Nevzat Arıcan, Elvire Servien, and Sébastien Lustig. *Methodology*: Christos Koutserimpas, Konstantinos Dretakis, Enejd Veizi, Nevzat Arıcan, Elvire Servien, Cécile Batailler, and Sébastien Lustig. *Project administration*: Konstantinos Dretakis and Sébastien Lustig. *Supervision*: Konstantinos Dretakis, Elvire Servien, Cécile Batailler and Sébastien Lustig. *Validation*: Christos Koutserimpas, Konstantinos Dretakis, Elvire Servien, Cécile Batailler and Sébastien Lustig. *Visualisation*: Christos Koutserimpas, Enejd Veizi and Nevzat Arıcan. *Writing–original draft*: Christos Koutserimpas, Konstantinos Dretakis, Enejd Veizi and Nevzat Arıcan. *Writing–review and editing*: Elvire Servien, Cécile Batailler and Sébastien Lustig.

## CONFLICT OF INTEREST STATEMENT

Christos Koutserimpas, Enejd Veizi and Nevzat Arıcan have nothing to declare. Konstantinos Dretakis: Consultant for Stryker. Elvire Servien: Consultant for Smith and Nephew. Cécile Batailler: Consultant for Smith and Nephew and Stryker. Sébastien Lustig: Consultant for Heraeus, Stryker, Depuy Synthes, Smith and Nephew. Institutional research support to Lepine and Amplitude.

## ETHICS STATEMENT

This study was conducted in compliance with the ethical standards established by the institutional and national research committees, adhering to the principles outlined in the 1964 Declaration of Helsinki and its subsequent amendments or equivalent ethical guidelines. Data collection and analysis followed the MR004 Reference Methodology from the Commission Nationale de l'Informatique et des Libertés (Reference No. 2229975V0). Additionally, the study received ethical approval from the Scientific Committee of Hygeia Hospital in Athens (Reference No. 663, 20/12/2023). Given the retrospective design of the study and the use of anonymized data, the requirement for formal patient consent was waived in accordance with institutional policies. As per institutional standards, formal patient consent is not required for this type of study.

## Data Availability

The data that support the findings of this study are available from the corresponding author, upon reasonable request.
